# Engaging with EPIO, a digital pain self-management program: a qualitative study

**DOI:** 10.1186/s12913-022-07963-x

**Published:** 2022-04-29

**Authors:** Katrine Bostrøm, Cecilie Varsi, Hilde Eide, Elin Børøsund, Ólöf B. Kristjansdottir, Karlein M. G. Schreurs, Lori B. Waxenberg, Karen E. Weiss, Eleshia J. Morrison, Elise Flakk Nordang, Audun Stubhaug, Lise Solberg Nes

**Affiliations:** 1 Department of Digital Health Research, Division of Medicine, Oslo University Hospital, Oslo, Norway; 2 Institute of Clinical Medicine, Faculty of Medicine, University of Oslo, Oslo, Norway; 3 Faculty of Health and Social Sciences, University of South-Eastern Norway, Drammen, Norway; 4 Faculty of Health and Social Sciences, Centre for Health and Technology, University of South-Eastern Norway, Drammen, Norway; 5 Norwegian National Advisory Unit On Learning and Mastery in Health, Oslo University Hospital, Oslo, Norway; 6 Department of Psychology, Health & Technology, University of Twente, Enschede, Netherlands; 7 Department of Clinical and Health Psychology, University of Florida, Gainesville, FL USA; 8 Department of Anesthesiology and Pain Medicine, University of Washington School of Medicine, Seattle, WA USA; 9 Department of Psychiatry and Psychology, College of Medicine and Science, Mayo Clinic, Rochester, MN USA; 10 Department of Pain Management and Research, Oslo University Hospital, Oslo, Norway; 11 Regional Advisory Unit On Pain, Oslo University Hospital, Oslo, Norway

**Keywords:** Digital, eHealth, Application (app), Chronic pain, Self-management, Engagement, EPIO, Qualitative study

## Abstract

**Background:**

Chronic pain conditions entail significant personal and societal burdens and improved outreach of evidence-based pain self-management programs are needed. Digital cognitive-behavioral self-management interventions have shown promise. However, evidence is still scarce and several challenges with such interventions for chronic pain exist. Exploring patients' experiences and engagement with digital interventions may be an essential step towards developing meaningful digital self-management interventions for those living with chronic pain.

**Objectives:**

This study aimed to gain insight into the experiences of people with chronic pain when engaging with EPIO, an application (app)-based cognitive-behavioral pain self-management intervention program.

**Methods:**

Participants (*N* = 50) living with chronic pain received access to the EPIO intervention in a feasibility pilot-study for 3 months. During this time, all participants received a follow-up phone call at 2–3 weeks, and a subsample (*n* = 15) also participated in individual semi-structured interviews after 3 months. A qualitative design was used and thematic analysis was employed aiming to capture participants’ experiences when engaging with the EPIO intervention program.

**Results:**

Findings identifying program-related experiences and engagement were organized into three main topics, each with three sub-themes: (1) Engaging with EPIO; motivation to learn, fostering joy and enthusiasm, and helpful reminders and personalization, (2) Coping with pain in everyday life; awareness, practice and using EPIO in everyday life, and (3) The value of engaging with the EPIO program; EPIO – a friend, making peace with the presence of pain, and fostering communication and social support.

**Conclusions:**

This qualitative study explored participants’ experiences and engagement with EPIO, a digital self-management intervention program for people living with chronic pain. Findings identified valued aspects related to motivation for engagement, and showed how such a program may be incorporated into daily life, and encourage a sense of acceptance, social support and relatedness. The findings highlight vital components for facilitating digital program engagement and use in support of self-management for people living with chronic pain.

**Trial registration:**

ClinicalTrials.gov NCT03705104.

**Supplementary Information:**

The online version contains supplementary material available at 10.1186/s12913-022-07963-x.

## Background

Chronic pain can impact physical, emotional and social functioning for the individuals living with pain, often resulting in significant personal and societal burden [1]. Multi-disciplinary care approaches, with the patient at the center of managing pain, are therefore increasingly recommended [2, 3], often pointing to the importance of raising awareness of the many physical, psychological and social aspects of pain, as well as encouraging and facilitating patient self-management, including planning and carrying out patients’ own goals [4].

For pain self-management, cognitive-behavioral therapy (CBT; centering around the relationships between thoughts, feelings and behaviors, and employing strategies to challenge and change thoughts and behaviors) [5], and acceptance and commitment therapy (ACT; focusing on acceptance of a situation and commitment to change as well as attention to own values) [6], represent the most recognized treatment approaches. CBT and ACT pain self-management interventions have for example been associated with improved psychological and physiological well-being, including improved quality of life, pain acceptance and self-efficacy, as well as reduced pain, anxiety and depression [7–9]. For various reasons (e.g., availability, geographical distance, personal preference and/or pain itself), such in-person treatment interventions are not always accessible to individuals living with chronic pain and new solutions and delivery options are needed [10].

Digital or electronic health (eHealth) solutions available via smartphones and tablets have potential for increased access and sophisticated strategies to help users implement self-management interventions into their everyday lives [11, 12]. Supporting this notion, existing digital CBT based interventions for self-management of chronic pain have shown potential to improve quality of life and support emotional well-being for people living with chronic pain [8, 13–15]. Studies have also shown existing pain management applications (apps) to be considered usable and liked by patients and health care professionals, however with a necessity for further research and scientific input [16]. Research examining existing digital self-management interventions is still at an early stage, with mixed findings and a number of identified limitations, including limited theoretical foundation [17], limited stakeholder (e.g., patients, health care providers) involvement in the development process [18], issues with attrition/adherence [19, 20], limited evidence of efficacy [12, 16] and limited implementation and actual use post study [21, 22].

A solid theoretical foundation of digital solutions is necessary in order to facilitate evidence-based treatment and support. Stakeholder involvement in the development of digital solutions is also essential to ensure that user needs and requirements are met [18, 23]. In order to strengthen probability of use, however, fostering and understanding user engagement in the actual use of these solutions is vital [24, 25]. Despite the importance of this aspect, there is still a lack of clarity about how engagement should be conceptualized within eHealth and digital solutions, and definitions range from psychological processes relating to user perceptions and experiences to actual intervention usage [25–27], the latter sometimes being the main, or only, conceptualization of digital user engagement. Researchers argue that the reasons why individuals choose to engage with eHealth interventions might be more important than the actual time spent using the intervention [26, 27], and have suggested that engagement should be conceptualized and specified within every new context used, and involve the user's behavioral as well as psychological relations to, or with, the digital program [27].

User engagement and adherence to an intervention, including intervention use as intended, raises the likelihood that the intended effect will be achieved [20, 23] and individual health-related goals reached [28]. The significant attrition, or disengagement, challenge occurring with eHealth solutions so far is therefore a major concern [19, 29, 30]. Seeking to address this issue, inclusion of persuasive designs that can motivate and engage users, even during difficult times, has been suggested [20, 30–32]. For example, there is evidence that design features such as personalization, praise, reminders and communication elements can foster user engagement and positive outcomes [19, 30–33]. To better understand engagement in digital solutions, however, psychological aspects should be thoroughly examined in addition to the frequently explored system use factor [27, 34]. This is particularly important to better understand the interplay between user perceptions, usage and effectiveness. Why do individuals engage in digital solutions, and how do they actively incorporate the intervention program into everyday life [35]?

The current research team designed and developed an app-based cognitive-behavioral pain self-management intervention called EPIO (i.e., inspired by the Greek goddess for the soothing of pain, Epione) [36–38] in response to some of the issues raised by existing research focusing on eHealth pain management programs. The EPIO intervention program is developed based on clinical and research-based scientific evidence, in close collaboration with key stakeholders, including people with chronic pain and related health care personnel [36–38]. EPIO's overall goal is to support self-management and well-being for people living with chronic pain in general (i.e., not condition specific pain). The current study builds on this research line.

### Objectives

This qualitative study aimed to explore the experiences of people with chronic pain when engaging with the EPIO intervention program. The exploration followed participation in a feasibility pilot-study where system use, perceived usefulness, ease of use and preliminary effects had been examined [39], and centered around gaining insight into the participants’ behavioral and psychological experiences and considerations related to the intervention program and their engagement with it.

## Methods

### Study design

This exploratory study reports on qualitative findings from follow-up phone calls and individual interviews conducted with participants in a feasibility pilot-study examining the feasibility and preliminary effects [39] of EPIO, an app-based pain self-management intervention program [36–39].

### Description of the EPIO intervention program

**Fig. 1:**
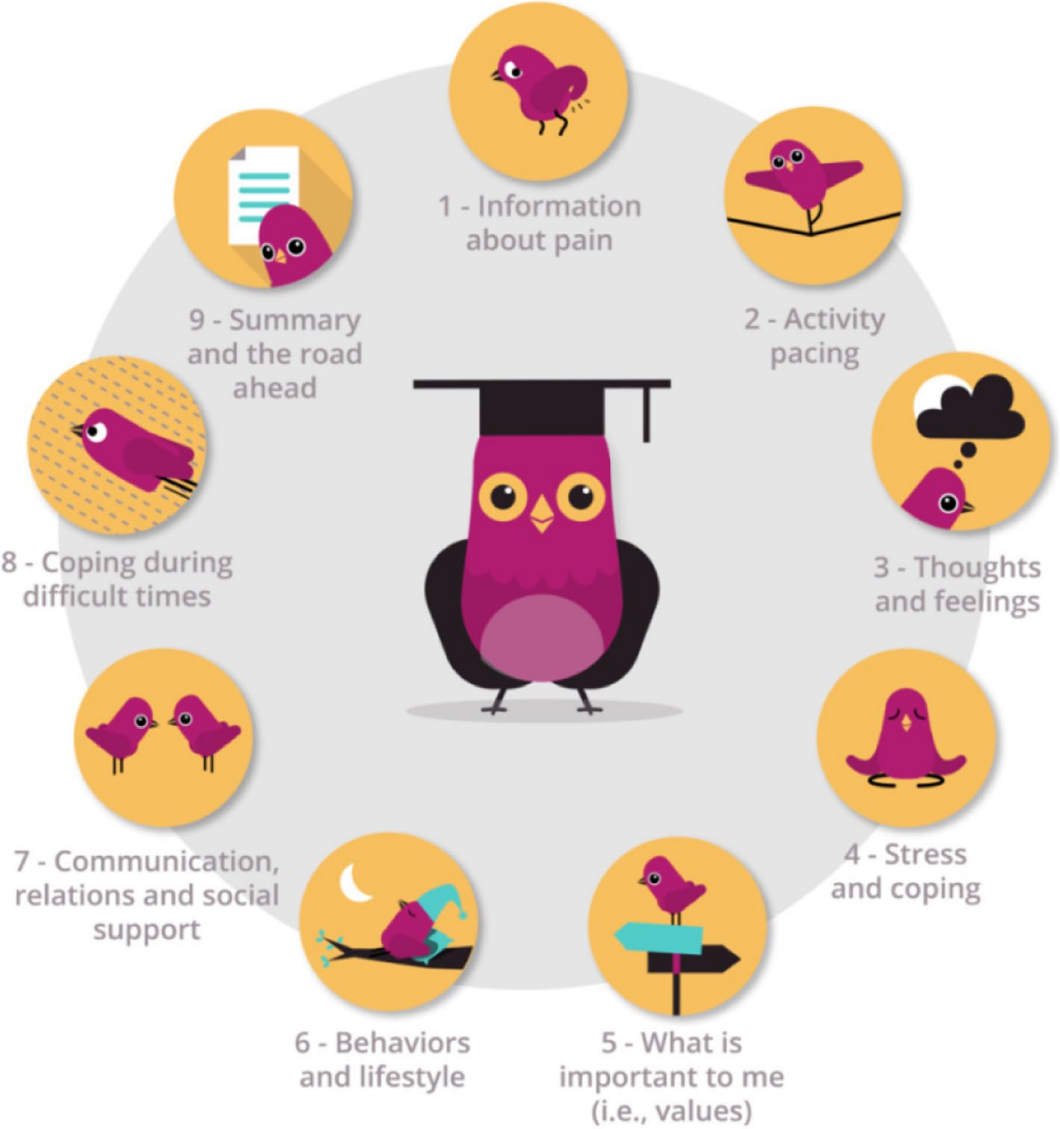
Overview of modules included in the app-based EPIO program

In the feasibility pilot-study, the EPIO intervention program was delivered in a simple blended care delivery model with one in-person introduction session, access to the app-based program, and one follow-up phone call [39]. The app-based program content is CBT-based with some aspects of ACT and consists of nine modules designed with several interconnected parts of educational information (e.g., coping strategies, thought challenges and the importance of activity pacing) and related exercises (e.g., diaphragmatic breathing, graded behavioral activation, mindfulness and progressive muscle relaxation) for people living with chronic pain. See Fig. 1 for an overview of the EPIO program.

The program also allows for daily tracking (i.e., individual registrations) of sleep, rest, activity level, pain and mood. To encourage program content practice, a 3-day delay occurs from completing each module until the next module opens (i.e., *practice-mode*). An animated avatar or “buddy”, a bird called “EPIOS”, accompanies and guides the user through the program. Details about the EPIO program, including the design, development and feasibility pilot-study testing processes, have been published elsewhere [36–39].

### Recruitment and participants

Recruitment for the feasibility pilot-study was conducted January—May 2019 [39]. Study and recruitment information was advertised through the research project website (www.epio.no), through the initiating institution (Oslo University Hospital), through social media channels, patient organizations’ websites and through collaborating partners (e.g., local health care services and primary care practices). Inclusion criteria were: age ≥ 18 years; living with chronic pain in general (i.e., not pain condition specific); pain duration ≥ 3 months (i.e., self-reported); access to a smartphone or tablet; being able to understand oral and written Norwegian; and being able to attend an in-person introduction session at a health care facility near the participants’ place of residence. Exclusion criteria included having an untreated severe mental illness, migraine, or cancer-related pain (i.e., all self-reported “yes”/”no” questions) [39].

A total of 50 people living with chronic pain participated in the feasibility pilot-study [39]. As part of the study procedure, all participants completed outcome measures at baseline and approximately 3-months follow-up, participated in an introduction session and received one follow-up phone call [39], during which participants were asked whether they would be willing to participate in an individual interview post-study. In order to ensure insight from a heterogeneous sample of the feasibility pilot-study participants, an inclusion matrix was developed where potential participants were selected for post-study interviews based on distribution of age (study range; 29–74) [39], gender (50/50), work-status (50/50) and program progress (i.e., even distribution of how many modules they had completed by 3-months feasibility pilot-study completion; all modules, 6 or more modules, or 6 or less modules). Based on this matrix, the research team then contacted relevant participants by telephone or text message to see if they were still willing to be interviewed and if so, to indicate a suitable time for the interview. The research team contacted 17 participants, of whom one declined to participate and one did not respond. A total of 15 participants were subsequently interviewed. Please see Table 1 for participant demographics.

**Table 1:** Baseline participant demographics

**Characteristics**	Feasibility pilot-study sample (*N* = 50)	Qualitative interview sub-sample (*n* = 15)
Age, mean (range)	52 (29–74)	52 (34–74)
Gender, n (%)		
Female	40 (80)	8 (53)
Male	10 (20)	7 (47)
Race, n (%)		
Caucasian	48 (96)	14 (93)
African American	1 (2)	
Asian	1 (2)	1 (7)
Employment status, n (%)		
Full-time/part-time work	14 (28)	6 (40)
Sick leave/disability benefits or retired	36 (72)	9 (60)
Program completion status at 3-months, n (%)		
Completed all nine modules	14 (28)	5 (33)
Completed at least six modules	31 (62)	5 (33)
Completed less than six modules	5 (10)	5 (33)
Years living with pain, n (%)		
1–3 years	10 (20)	2 (13)
3–5 years	3 (6)	1 (7)
5–10 years	13 (26)	6 (40)
> 10 years	24 (48)	6 (40)

### Data collection

#### Follow-up phone calls – during intervention

The participants in the feasibility pilot-study received a follow-up phone call from a member of the research team at 2–3 weeks after the introduction session to see how things were going and inquire about any questions or comments regarding the use of the EPIO intervention program [39]. The phone call lasted approximately 10–15 min and the research team member making the call took notes during the conversations.

#### Semi-structured interviews – post-intervention

Interviews were conducted post follow-up outcome measure completion in the feasibility pilot-study [39], at approximately 3-months. The individual, semi-structured interviews lasted 30–40 min, were conducted by telephone by a member of the research team and were audio-recorded and transcribed verbatim*.* A semi-structured interview guide (see Additional file 1) was used to guide the interviews. Themes explored were related to engagement and included behavior (e.g., actual use), motivation (e.g., program content practice), design features (e.g., animated avatar, rewards and trophies, individual registrations, and reminders), communication (e.g., involvement of healthcare professionals in use of the EPIO program, and their perceptions of in-person support such as follow-up phone calls), and perceived value and satisfaction with the EPIO intervention program.

#### Ethical approval and informed consent

The EPIO feasibility pilot-study, including the data material collected in the current study was approved by the Regional Committee for Medical and Health Research Ethics (REK 2018/8911) and the Oslo University Hospital Institutional Review Board equivalent function (PVO 2017/6697) [39]. All participants provided written informed consent.

### Data analysis

The data material (i.e., notes from follow-up phone calls and interview transcripts) were uploaded to the software program NVivo version 12 (QSR International, Victoria, Australia) and analyzed using Braun and Clarke's [40] six-step thematic analysis approach to capture user experience themes with a codebook approach analysis [41]. The data material from the two sources (i.e., method triangulation) was merged and treated as one material in the analysis process, but could still be looked at separately as the transcripts were uploaded to NVivo in two colors. The first step involved the first author (KB) listening to the audio-recorded interviews while taking notes, then reading the transcribed interview data and the notes from the follow-up phone calls while taking additional notes. In line with an inductive approach, within step two, codes were derived from the data, extracting embedded meanings in the sentences. The first and second authors (i.e., KB, CV) then reviewed and compared the codes in step three and categorized them into themes. In the fourth step, main themes and subthemes were compared with the codes and refined to ensure coherence before comparing with the entire data material to ensure representation. In the fifth step, the extent to which data from the two sources were included in themes and sub-themes was assessed, and data from both sources were deemed to be represented in all themes and sub-themes. Then, in the sixth step, the themes and subthemes were thoroughly reviewed and compared (i.e., researcher triangulation) within the core research team (i.e., KB, CV, LSN and HE), before final results were written up in step seven. In the final step, quotes were selected, primarily from the interviews due to material richness, to illustrate the analysis and ensure credibility and transparency.

## Results

**Fig. 2:**
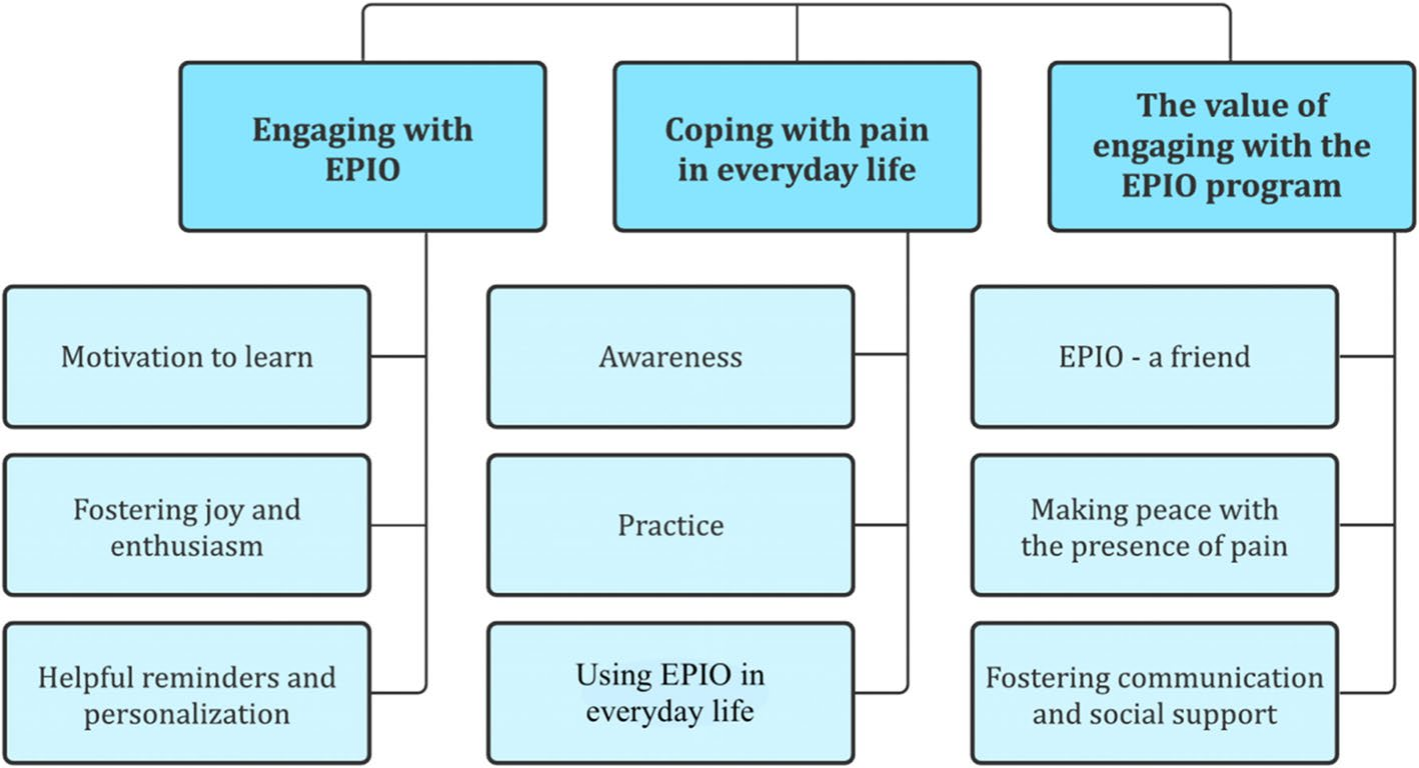
Identified main topics and sub-themes, related to participants' experiences when engaging with the EPIO intervention program

The findings from the thematic analysis provided insight into participant's experiences when engaging with the EPIO intervention program and were identified and organized into three main topics: (1) Engaging with EPIO, (2) Coping with pain in everyday life, and (3) The value of engaging with the EPIO program. Please see Fig. 2 for an illustration of findings.

### Engaging with EPIO

The participants described EPIO as presenting a new way to learn pain self-management, with valuable content and design features, and options for encouragement and support while facing the challenges of daily life with pain. Aspects described as important when engaging with EPIO were sorted into three sub-themes; motivation to learn, fostering joy and enthusiasm, and helpful reminders and personalization.

#### Motivation to learn

Participants described being motivated to participate in the study and use the EPIO program because they were curious and willing to try something new that might help with their pain. They reported wanting to learn how to better manage their pain, without the use of pain medication, for example through stress management and relaxation exercises, and described their motivation as being able to take control of their situation and feel better about themselves, with hope for improved well-being:

*“My motivation is to try to get control of my pain. I have promised myself that I will try everything to overcome the pain, and if I do not become pain-free, then I will at least get control of it and improve my quality of life. That's what's in my head. So, my motivation is really that I am to feel better about myself. And I have read a lot in recent years, and have concluded that there are many things one can do to decrease the pain, without using medication. And a lot of it concerns stress and relaxation”. (Interview, participant 5)*

Another type of driver for motivation to engage and learn was the availability and easy access of EPIO. Some compared the accessibility and sophistication of the EPIO program to a subway ticket program they could have on their phone, to use whenever needed or whenever they felt like it:

*“I look at the phone, there’s my subway ticket, so then I have to bring my phone, and then I also have access to EPIO any time it suits me, I think that’s great”. (Interview, participant 2)*

#### Fostering joy and enthusiasm

Participants described the EPIO program, its content and design features as engaging and motivating for use. They used words such as: excited, happy, surprised and hopeful when talking about the program and some even compared EPIO to a gift:

*“I’m so excited about the app – it’s amazing! It’s like a Christmas present – I’m eager and always looking forward to opening it”. (Interview, participant 12)*

Participants also expressed their enthusiasm regarding EPIO's avatar elements, such as the animated bird, EPIOS, and described EPIOS as funny, engaging, and with useful quotes of wisdom, reporting perceiving EPIOS as a good companion and friend throughout the program:

*“It's [EPIOS] so cute. It looks happy. All the great wisdom it’s sharing and everything, super”. (Interview, participant 1)*

Some participants did however express mixed feelings, stating that the bird appeared too often and that while joyful and fun, EPIOS could also be a tad annoying. Other participants, based on previous experiences with gaming, suggested having even more “fun” parts included in the program to enhance engagement.

The obtainable rewards and trophies in EPIO, aiming to praise, were perceived as fun and motivating for use and participants described being excited when receiving a trophy, such as the “master of practice” trophy. Others, however, referred to the rewards and trophies as neither motivating nor discouraging for their program engagement, instead describing finding enthusiasm and joy, a reward in itself, in mastering an exercise or completing aspects of the program.:

*“The reward for me is to feel good afterward, so ... yes. Of course, it feels good to complete a chapter, it does. But that feels good in and of itself, so I do not need trophies. Then, again, people probably differ”. (Interview, participant 49)*

#### Helpful reminders and personalization

Participants expressed wanting to engage with EPIO, but reported sometimes finding it challenging to use the program when work or everyday life became stressful or hectic, stating that setting aside time or space to engage with the program could be challenging. Participants did however describe the *reminde*r function in EPIO as very helpful, stating that being reminded, for example to do reading or exercises at a specific time, could be a highly useful feature:

“*... but I easily forget to use it so reminders are nice, have to get it into everyday life”. (Interview, participant 34)*

Participants also expressed appreciating the option to personalize the content and mark topics and exercises as *my favorites*, making their favorite aspects of the program more accessible to them. They also described appreciating being able to change their favorite content when desired, as interests and needs changed throughout the program. A few of the participants also expressed a wish to be able to receive push alerts related to their favorites, and described wishing for personal or customized built-in messages such as: “*how are you feeling today?”* and *“here is one of your favorite exercises”.*

The options for personalization were described as valued features, including the option for daily individual registrations (i.e., sleep, rest, activity level, pain or mood), as participants stated that it could be easy to forget previous mood or activities (e.g., what you did yesterday) when living with chronic pain. None of the participants, however, described discovering significant variations in everyday life based on their personal registrations in EPIO. Some described not really caring one way or other about the personal registrations, while others stated that such registrations would even represent a negative focus for them:

*“Every time I got those registrations, it's like ... you try to tell yourself that you sleep well every night, you feel good, and things like that ... But then it's like you have to start to notice, and feel, that you are not actually doing so well after all”. (Interview, participant 12)*

### Coping with pain in everyday life

Participants described the potential role of EPIO, when aiming to cope with pain in everyday life, as helping increase awareness about pain and the impact of pain, stress and pain self-management, highlighting the need to practice content and exercises often to achieve the desired effect, and pointing to EPIO as something to lean on when dealing with the daily challenges of living with pain. Topics raised were sorted into three sub-themes; awareness, practice and using EPIO in everyday life.

#### Awareness

Participants described how their engagement with the EPIO program had been meaningful to them, gaining new hope of being able to cope with pain and of living a life with pain, better than before. Many of the participants described increased self-awareness of own condition, and of the little things that one can do to make oneself feel better, rather than focusing on the things that one cannot change:

*“Awareness, that's the best word I can use to describe it. Awareness that there are things that can make you feel a little bit better. And it's all about doing things, and not doing things, if you know what I mean?” (Interview, participant 17)*

Diaphragmatic breathing was described as something that had always been a challenge, and participants stated that the EPIO program had provided them with increased awareness of what pain and stress could do to their body, and reminded them of the importance of knowing how to calm down through focusing on breathing and deep diaphragmatic breathing. Breathing and relaxation exercises were even described as the participants favorite parts of the program:

*“It has given me exercises to help reduce stress a bit. To let things go, that it's not so important, perhaps, and to breathe with your stomach”. (Interview, participant 49)*

During the follow-up phone calls and interviews, several participants mentioned that they were already familiar with the type of content found in EPIO, and that they had not necessarily learned a lot of new material. They did however emphasize that the program raised awareness and served as a valuable reminder of all the strategies that can be used to cope with the pain, for example finding a good balance between activity and rest, and being kind to oneself:

*“Yes. I think it has been useful in the sense that it increases understanding. Really of what all health professionals you meet say is important, that you have to take small steps and that you have to find a balance between rest and activity ...” (Interview, participant 24)*

#### Practice

Participants described EPIO as a valuable tool for self-management of chronic pain. They did however highlight the need to practice program exercises often in order to achieve the desired effect, regardless of whether one experienced pain or whether life was hectic:

*"Yes, but then I will be the first to say that then you have to practice, much more than what I have done. Because then you have to, I believe you have to be very determined and use these breathing exercises and relaxation methods repeatedly. Preferably several times a day, if you get to it, and that is my opinion". (Interview, participant 34)*

Regarding the 3-day delay between modules in order to encourage practice, most participants stated that they understood the value of this *practice-mode*. They did however express mixed feelings, describing having been somewhat impatient in the beginning, but then experiencing that not rushing through the program made them feel better. Changes in thoughts and attitudes towards the concept of practice were also described in this process:

*“I think it was very good to get to know the exercises ... or the topic at hand. Because then you were forced to go back, and then the exercises that were not so ... well, that did not appeal to you at first ...and then wow, they were great, right? So I actually think I could have missed a lot, so I think it was very nice”. (Interview, participant 4)*

#### Using EPIO in everyday life

Participants described their use and involvement in EPIO as structured, with a daily routine. The most important factor was described as being able to have something to lean on when dealing with the pain, and some stated that they preferred to do EPIO exercises in the morning as a way to “wake up tired bodies” and get a positive start to their day, others stated that they enjoyed engaging in relaxation exercises in the evening, while others again reported doing both:

*“I think it's very nice to use it in the morning. So that I, in a way, can lean on it, on days when I struggle as the day progresses. That I sort of carry it with me in the back of my mind”. (Interview, participant 4)*

Being at home was described as a favorite place for using EPIO and practicing the material, while a few reported having used EPIO at work during a break, or when traveling. Participants described liking being able to choose between reading and listening, stating that their preferences depended on the situation they were in at the time of practicing. For example, if they wanted to relax, they described it as preferable to lie down on their bed and listen. Participants also reported being able to concentrate, in quiet surroundings, as very important when engaging with EPIO:

*“I have kind of mainly been at home. That is where I, in a way, can be mostly left alone and do it, in a way, on my own. Because when all of this was new, I did not know what would be next, so I have, in a way, consciously used it at home to be able to concentrate”. (Interview, participant 36)*

### The value of engaging with the EPIO program

Having described central aspects of engaging with EPIO and ways in which EPIO could be used to cope with pain in everyday life, the participants also described additional values of engaging with the EPIO program, including potential benefit of use for many, not just people living with pain. EPIO was also described as a potential “friend” in day-to-day life, and as having the potential to enhance understanding and self-acceptance for people living with pain, as well as aiding with communication and social support. Topics were sorted into three sub-themes; EPIO—a friend, making peace with the presence of pain, and fostering communication and social support.

#### EPIO – a friend

When asked if they would recommend EPIO to other people living with chronic pain, most participants said yes, and also stated that they believed that people in general, not just those living with chronic pain, could benefit from using the EPIO program:

***“****Well… It kind of, deals with the whole person. With emotions and social network and work tasks and ... well, the total, overall picture. Instead of just focusing on where it hurts right there and then”. (Interview, participant 2)*

The participants displayed vulnerability in the interviews, sharing information about their relationships and how it can be very challenging to communicate with friends and family members about their pain. Describing it as easier to withdraw than to talk to people, they stated that living with chronic pain entails a major risk of becoming isolated and uninvolved in social activities. EPIO was as such, by some of the participants, perceived as “a friend when in need”. They described listening to the voice of the EPIO program, a voice that was perceived as calm and pleasant, and feeling almost as if having a real human friend:

*“If you are in a lot of pain, then you are, in a way, quite alone. And if you are not so good at sharing, describing how you are feeling to others. It's not so acceptable or popular to say that you are in pain and ... well, that things can be a little tough sometimes. People would rather not hear about such things. I have felt that you can withdraw and listen to the soothing voice, and what you can do ... it has given me a break……, and it has provided relaxation”. (Interview, participant 13)*

#### Making peace with the presence of pain

Participants described EPIO as having provided a better understanding of their own situation living with pain, facilitating self-acceptance, perhaps even pain acceptance. Reporting consequently having gained a sense of confidence, the participants expressed a need to make peace with the presence of pain:

*“What is useful with EPIO is to become more ... to achieve a degree of inner peace, in relation to my pain. These are things like ... I will never get rid of my pain, so I have to … rather than arguing with it all the time, standing on separate sides of a courtyard, fighting, to be a little more in tune. So, becoming better friends with the pain sort of, and work with the pain, not just against it”. (Interview, participant 4)*

Participants also reported recognizing the importance of prioritizing themselves and setting aside time for themselves, although recognizing that this might be easier said than done:

*“I have learned to relax; when I have the time (laughs a little). And I have learned various breathing exercises and relaxation methods that I was not familiar with before. And I have learned that you have to take the time, in a stressful everyday life. Even if you are at home, sick, and in pain, you have to prioritize yourself. It is of course so easy to say - not so easy to do”. (Interview, participant 34)*

#### Fostering communication and social support

When asked what they thought about receiving a follow-up phone call during the study, participants reported appreciating being able to talk to a member of the research team, someone who had experience with and/or knowledge about the EPIO program and its self-management strategies. Despite the brief nature of the phone call, they reported feeling the research team cared about them, showing interest in their well-being, and a few of the participants stated that having lived with pain for a long time, it is “rare to receive personal follow-up”:

*“For me personally ... well, you are a bit left to yourself in the health care system. Here, there’s actually someone calling. If there is anything my, if we can call it that, patient group needs, it’s often to talk to someone. Regardless of what it is. And especially someone who has a certain understanding of what it is you are doing and what it is you are going through”. (Follow-up phone call, Participant 17)*

A few of the participants reported having used the EPIO program together with their provider (e.g., physiotherapist, psychologist), in group therapy at a rehabilitation center, or with other people who also live with chronic pain. Having shared the use, or experience of use, was described as positive and working with EPIO described as easier due to having the support of another person:

*“So, using the app that way is ... and hearing the experiences of others, people I know, who use it, if you know what I mean, it has been very helpful. And then we can discuss and talk about how we… how do you use it, how do I use it. Oh, you think about it that way, and then we go through it a bit….where the information about…. getting to know the pain, and stuff like that, so how we choose to interpret it. That has been very useful”. (Interview, participant 10)*

Participants also mentioned that family members and friends should consider engaging in EPIO to gain insight into how it is to live with chronic pain, maybe attaining a better understanding and becoming more supportive or at least more informed:

*“The advantage with that is that you can get an understanding of people who may struggle, even though it’s not visible to others. That one becomes more generous perhaps, and more understanding with regards to that part”. (Interview, participant 1)*

## Discussion

Participants living with chronic pain described experiencing motivation to learn new ways to self-manage pain after engaging with EPIO, and reported feeling enthusiastic, encouraged and supported by the program. They also reported being able to incorporate the content of EPIO in support of daily coping, stressed the importance of practice, and portrayed the program as facilitating increased awareness of the many aspects of living with pain. EPIO was also described as being a potential “friend”, fostering acceptance, improved communication and social support, and was referred to as something people in general, not just those living with chronic pain, could benefit from using. The following sections elaborate on these aspects and how an eHealth self-management solution such as EPIO can engage and be of potential value for people living with chronic pain.

### Motivation for engagement

Participants described desire to learn and try something new to better manage their own pain as the main motivation for engaging with the EPIO program. They expressed motivation to take control of their own lives, and wanting to have something, such as coping strategies, to lean on when dealing with the presence of pain in everyday life. These findings are in line with existing research stating that people's desire and motivation to self-manage pain are often related to their goals and values, and that engaging with self-management programs is a valued way to maintain a sense of control over life [42, 43].

Digital solutions allow users to control when, where, and how they engage with a program or intervention [44, 45], and the availability and easy access to the EPIO program (i.e., via smartphone or tablet) was highlighted by participants as a motivating factor for engagement. They also emphasized the program content (i.e., educational information and related exercises) as engaging, beneficial and of great value to them, displaying personal initiative and self-guidance with the EPIO program content. This is also in line with existing research showing that when users can identify themselves with the self-management program, their engagement increases [46].

The effect of self-management interventions likely stands or falls with practice [47, 48], and while participants in the current study expressed finding it challenging to prioritize program use during hectic periods of time, they recognized the need for practice and described appreciation for the EPIO program reminder functions, and options for personalization of content, to help them achieve the desired effects. Along the same line, participants described finding the EPIO program engaging and motivating for use, fostering enthusiasm and feelings of joy, excitement and interest. Such positive emotions may be a motivator for change, engagement and exercising personal control over one’s situation [49], potentially even fostering continued use. As design features may enhance or regulate people`s emotions, behavior and choices regarding self-care [30, 33, 50], affective design feature experiences may even play a role in achieving digital intervention effectiveness [19].

Aiming to praise and motivate for engagement and use, the EPIO program design contains obtainable rewards and trophies [37]. However, participants in the current study described mastering exercises and completing program steps as their primary rewards. When people are involved in opportunities that allow for personal initiative and self-guidance, as with the EPIO program, autonomous motivation can thrive, and they are more likely to feel interested and engaged [42]. Self-determination theory [51, 52], centering around motivation based on fulfilling needs for competence, relatedness and autonomy, may hence also shed some light as to why intrinsic motivation seemed more apparent than external factors (e.g., praise and rewards) in this study. When people are more motivated by their values, interests and enjoyment of behavior change, they tend to stay continually engaged in the self-management process and be more satisfied [42, 43]. This can also be of interest for the eHealth adherence/attrition conundrum [17, 27, 28].

Stakeholder involvement has been employed throughout the design and development phases of the EPIO program [36–38] to ensure vital input for program use, usefulness and usability, as well as for adherence/attrition related aspects of the program [18]. Requests for daily registration options (e.g., sleep, rest, activity, mood and pain) were originally requested by end users (i.e., people living with pain) [36], and incorporated into the program [37]. Participants in the current study did however not perceive daily registrations as particularly helpful, even describing them as demotivating. The pain registration variable was subsequently removed from the EPIO program post feasibility pilot-study in preparation for the randomized controlled trial, while the other variables, potentially less impeding on pain self-management, still remain.

### Digital pain management in everyday life

Several factors may influence how well people use strategies in a self-management program and how long they stay engaged. For example, self-efficacy, or one’s belief that one can utilize the self-management techniques, may play an important role in this type of engagement [43, 53] and as several participants in the current study described an improved sense of control and self-confidence from the use of EPIO, this may indicate a potential digital self-management—self-efficacy link to be further explored.

Living with chronic pain entails a multitude of challenges [1] that can make frequent and continuous use of self-management programs and tools complicated and arduous. In the current study, participants reported family issues, time constraints and illnesses as the main reasons when and if they struggled to engage with the program, all known barriers to the use of digital solutions as well as self-management programs in general [25, 48, 54]. However, having attained an increased awareness and experienced desired benefits of at least parts of EPIO (e.g., relaxation exercises), participants also reported being aware of the potential negative impact of stress on pain, breathing and the “mind–body” connection. This awareness appeared to encourage participants to deal with obstacles in new ways and to continue using the EPIO program, for example seen in the perceived importance of breathing exercises as valuable tools for everyday management of stress and pain, a finding also in line with existing research [55–57].

### Additional values engaging with digital pain management interventions such as EPIO

Participants in the current study referred to EPIO as having the potential to also help valued people (e.g., friends, family) gain a better understanding, perhaps subsequently becoming more supportive, of those living with chronic pain. This finding is in line with existing research indicating that people's desires and motivation to learn self-management of chronic pain also can entail a need for connection and a sense of belonging (e.g., relatedness) [42, 43, 48]. Similarly, the sharing of EPIO use and experiences with others seen in this study is consistent with existing research indicating that app-based solutions may facilitate valuable discussions between patients and their health care personnel [58].

EPIO was also described as a friend, allowing participants to feel less alone living with pain. This suggests facilitation of a supportive interpersonal aspect, and is also consistent with engagement-related research indicating that users are often willing to engage more and create relations with intervention technology that share features similar to human relationships [59, 60], and such human-technology relationships may increase the perceived meaningfulness of digital interventions [60] and play a role in fostering user engagement [61].

The complexity, demands and challenges of living with chronic pain may lead to a draining of the capacity to self-regulate, that is regulate own thoughts, emotions and behavior [62–64]. Participants in the current study reported that EPIO provided them with a better understanding of their own situation, promoting acceptance. Participants also described having realized the importance of prioritizing their own needs and goals in this process, which again allowed them to focus on self-management and self-regulation. Self-regulatory capacity may play an important role in terms of ability to engage and undertake the necessary day-to-day practice in a digital self-management setting, and continuing to find ways to help people with chronic pain build or strengthen their self-regulatory capacity and support motivation to engage in pain self-management strategies, such as the EPIO program, appears vital [65, 66].

Finally, there are some indications that eHealth programs may yield better adherence and positive outcomes when combined with in-person support [18, 67–69]. The EPIO intervention program was therefore delivered in a blended-care model (i.e., one in-person introduction session, nine app-based modules, and one follow-up phone call) to support adherence and user engagement. Participants in the current study reported appreciating the contact, albeit limited, with the research team, valuing being able to ask questions concerning their use of EPIO and describing generally feeling engaged, supported, and taken seriously. The findings also point to the difficult aspects experienced by many people living with chronic pain; the feeling of being alone and not experiencing being heard, seen or understood [70]. Could digital self-management solutions, such as EPIO, delivered in a simple blended care model entailing some, although minimal, contact with providers, contribute to alleviating some of these substantial challenges for people living with chronic pain?

### Strengths and limitations

The current study has a number of limitations. First, the participants in the feasibility pilot-study were recruited through social media and collaborating partners, and it may therefore be assumed that the participants were highly motivated for participation. However, ensuring a balanced group of participants in the interviews (e.g., gender, work status and program progress) allowed insight into a range of participant perceptions and experiences. Second, the first author was involved in analyzing the transcribed data, including creating the coding framework and the organization of themes, which could introduce a risk of researcher bias in the qualitative evaluation. The study did however aim to address this potential bias and assure transparency of data by the inclusion of co-authors and the core research team in the analysis process. Third, of the 50 participants in the feasibility pilot-study, 28% completed all 9 program modules, 62% completed six modules or more, and 10% completed less than six modules by the 3-months study completion (see Table 1 for details). Even though the EPIO program can be completed in 27 days (3 days × 9 modules), however, the goal is not for the participants to complete the program as quickly as possible, but rather spend time practicing the content before moving on to the next topic. This is underlined by the EPIO program and was also stressed by the project team, and it is therefore possible that a 3-months study period is not enough time for people with chronic pain to complete such a program. In the current study however, the 15 participants interviewed were selected to be representative of age, gender, work-status and program progress (i.e., even distribution based on number of modules completed) from the feasibility pilot-study, and module completion progress (i.e., all modules, ≥ 6 modules, and < 6 modules) was evenly distributed between included participants (i.e., 33% each, see Table 1).

Also, the study sample was primarily Caucasian and the feasibility pilot-study participants primarily female, limiting the transferability of these findings, and given that women primarily volunteered for study participation, the feasibility pilot-study may be considered a sample of convenience. The prevalence of chronic pain is however higher among females compared to males [71], and self-management interventions also appear to be a preference for females compared to males, at least when it comes to study recruitment and participation [72]. In the current study, a fairly balanced number of females (53%) to males (47%) were included in the interviews, and all participants' input was assessed together during the analysis, aiming for a varied, rich insight into people’s perspectives when engaging with EPIO.

This study also has a number of strengths as aspects of trustworthiness [73] (e.g., credibility, transparency) were well covered. First, the interviews were conducted by research personnel who were not involved in the data analysis, reducing potential research bias. Second, the credibility was assured by describing all steps in the analysis process as thoroughly as possible to allow the reader to follow the logic of the findings. By embedding carefully chosen quotes in the final manuscript, the researchers aimed to give the participants a voice in the outcomes while contributing to the credibility and transparency of the research. Trustworthiness was also ensured by using methods triangulation including combining data from two sources (i.e., notes from follow-up calls and interviews transcripts) and researcher triangulation (i.e., involving several researchers in the study [74]. The latter was done to reduce the potential bias that comes from a single researcher doing the analysis alone.

### Future implications

The current study sought to address gaps in the existing literature examining engagement with digital pain self-management interventions. Identified factors of engagement included motivation to learn, design and content fostering joy and enthusiasm, as well as helpful reminders and options for personalization. Such findings are transferrable to other digital interventions, including non-pain related self-management contexts such as for example behavior changes interventions, weight-, and stress-management interventions, and medication management and adherence programs. That positive emotions such as joy and enthusiasm can motivate people to engage in self-management and care supports existing research [49], and future research should examine how positive affect can be strengthened and channeled to further foster engagement with digital solutions. Future explorations of these aspects may also help understand why certain design features (e.g., gamification) work for some but not for others [27, 75]. These are all aspects that may be of interest in research focusing on digital health interventions, regardless of end-user population or context.

Even though the EPIO intervention examined in the current study targets chronic pain in general (i.e., not condition specific pain), it should be emphasized that “chronic pain” is rarely considered a homogeneous entity, and type, form and degree of pain may differ depending on condition (e.g., fibromyalgia, spinal cord injury, trigeminal neuralgia). EPIO is developed based on recognized CBT/ACT related approaches to general pain self-management, and a degree of beneficial impact could therefore potentially be expected regardless of pain condition type. Considering the heterogeneity of chronic pain, however, future research should strive to explore potential impact depending on pain condition(s).

The nature of self-management processes is complex, and social relations and support may be particularly important for these processes [76]. The indications that digital programs may foster communication and social support, subsequently impacting engagement, as indicated in the current study, should therefore be further explored. These are also aspects that will be of interest and transferable, regardless of context. For example, research already states that peer support can improve engagement with digital self-management or cope with a chronic condition in general (e.g., asthma, diabetes) [77]. With the suggestions of improved sense of control and self-confidence following use, future studies should also explore the role of self-regulation and self-efficacy when engaging with such digital self-management interventions. Furthermore, given the significant adherence/attrition challenges with digital interventions [25, 27, 28, 78], exploring ways to further cultivate motivation and engagement and aid people with chronic pain overcome barriers for use of effective digital self-management programs appears crucial.

It is possible that the simple blended care delivery model employed may have impacted motivation for engagement in the current study. Future research should therefore also aim to explore engagement comparing delivery models (e.g., app-based only, simple blended care, more complex blended care etc.) of digital pain self-management interventions. Finally, the current findings showing program engagement through motivation to learn, enthusiasm and use of personalization, as well as raised awareness and a sense and fostering of support, compliments existing feasibility (e.g., use, usefulness, ease of use) findings for the EPIO program [39]. Establishing efficacy, a frequent limitation for existing eHealth pain management programs, should be a goal for future research examining digital self-management interventions, and a randomized controlled trial testing the efficacy of EPIO is currently being conducted.

## Conclusion

This qualitative study explored patients' experiences and engagement with EPIO, a digital self-management intervention program for people living with chronic pain. Findings identified valued aspects related to motivation for engagement, showed how people with chronic pain may incorporate such programs into everyday life, and also how such a program may encourage a sense of acceptance, social support and relatedness. These findings highlight vital components encouraging digital program engagement and use in support of self-management for people living with chronic health conditions, such as chronic pain.

## Supplementary Information

**Additional file 1: Appendix 1.** Semi-structured interview guide

## Data Availability

The datasets generated and analyzed during the current study are not publicly available due to national and organizational privacy and security regulations, but can be made available from the corresponding author on specific request, if approved by the hospital Department for Data Protection and Information Security at Oslo University Hospital.

## Abbreviations

ACT: Acceptance and Commitment Therapy
App: Application
CBT: Cognitive Behavioral Therapy
eHealth: Electronic health
