# Supplementary material for: Engaging with EPIO, a digital pain self-management program: a qualitative study
Source: BMC Health Serv Res. 2022 Apr 29;22:577. doi: 10.1186/s12913-022-07963-x (PMC9052507; doi:10.1186/s12913-022-07963-x)
Supplement: Supplementary file 1 — Additional file 1: Appendix 1. Semi-structured interview guide [file 12913_2022_7963_MOESM1_ESM.docx]

Interview guide

Thank you for helping us tests the EPIO pain self-management program!

I will put you on speaker when using a tape recorder during this interview; can you hear me all right?

I will now ask you some questions about how you have experienced the EPIO app, experiences you have made during the intervention program and any suggestions you might have for improvements. The interview will last approximately 30 minutes, but please feel free to stop me along the way if you have questions or need a break.

# Questions

## Generally

- Please explain your experiences using the EPIO app? (elaborate)
- How often have you used the EPIO app?
- When in the day did you prefer to use the EPIO app?
- Where did you use the EPIO app the most? (e.g., travel, treatment, home, and the reason for this?)
- Have you involved healthcare professionals in your use of the EPIO app?
  - If yes, can you tell me more about it?
- Has the EPIO app been useful?
  - What has been particularly useful?
  - What has not been so useful?
- Have you learned anything new about pain and pain management that you did not know before?
  - If yes, can you tell me more about it?

## Contents

- How many of the topics have you been through?
- What do you think about the content? (e.g., theme/quantity)
- What do you think about the information material? (Only if they have not said anything about this)
  - Useful/not useful?
- What do you think about the exercises? (Only if they have not said anything about this)
  - Useful/not useful?
- What do you think about the personal registrations (Only if they have not said anything about this)?
  - Useful/not useful?
  - How often have you used them?
  - Why/why not?
- Did you go through any of the content/exercises several times?
  - If yes: Why?
  - If no: Why not?
- If the participant has been through and used many/all topics: What was it that motivated you to use the app, practice/do the exercises again?
- Were there any exercises that worked better than others? (why)
- If the app was not much used: What could motivate you to use the app more? What could motivate you to repeat exercises?
- What do you think of the bird EPIOS that comes with suggestions and summaries along the way?
- What do you think about the quotes?

## Functionality

- Have you had any technical problems along the way?
- What do you think about the settings options? (e.g., recordings, reminders, sound)
- Did you prefer reading or listening to the content of the topics? (e.g., elaborate on why?)
- What do you think about the voice and the tempo in the audio files of the app?
- Would you prefer to be able to automatically move from one step to the next when listening to the app, or be able chose when to move on by yourself?
- What do you think about having access to a new topic after three days?
- What do you think about the opportunity to mark different steps and exercises as favorites?
- If it was possible to use the app on more than one device (e.g., mobile as well as tablet), do you think you would use it on more than one device? Why? Why not?

## Design

- How easy do you think it was to navigate and follow the intended steps of the app? (What do you think about the overview in the app?)
- How easy or difficult do you think it was to follow the topics for each step?
- What do you think about the pictures and images included in the various themes?
- How did you experience the app icons? (e.g., notebook, chair, bed, headphones)
- What do you think about the font size?
- How did you experience the rewards that appeared as you progressed?

## The pilot study

- Do you have any comments related to the introduction meeting?
  - What was it like to install the app?
- How did you experience receiving a phone call from someone in the project after 2-3 weeks?
- What was it like to answer the questionnaires/outcome measures?
- Did the questionnaires enable you to give a good picture of how everyday life and life with pain have been for you during this period?
  - If not - Do you have any suggestions related to how the questionnaires could help you provide a better picture of what is actually your daily life and reality?

### Closing questions

- What did you like? (name 3 things)
- What would you change? (name 3 things)
- Was there anything you missed?
- Would you recommend EPIO to others?
  - If yes, what would you say to them?
  - If no, would you like to say something about why?

• Do you have any other feedback/input for us?

• Finally, overall, what has using the EPIO app meant or provided for you?
